# Nucleosome binding by the pioneer transcription factor OCT4

**DOI:** 10.1038/s41598-020-68850-1

**Published:** 2020-07-16

**Authors:** Kenta Echigoya, Masako Koyama, Lumi Negishi, Yoshimasa Takizawa, Yuka Mizukami, Hideki Shimabayashi, Akari Kuroda, Hitoshi Kurumizaka

**Affiliations:** 1 Laboratory of Chromatin Structure and Function, Institute for Quantitative Biosciences, The University of Tokyo, 1-1-1 Yayoi, Bunkyo-ku, Tokyo, 113-0032 Japan; 2 Department of Biological Sciences, Graduate School of Science, The University of Tokyo, 1-1-1 Yayoi, Bunkyo-ku, Tokyo, 113-0032 Japan; 3 Graduate School of Advanced Science and Engineering, Waseda University, 2-2 Wakamatsu-cho, Shinjuku-ku, Tokyo, 162-8480 Japan

**Keywords:** Nucleosomes, DNA-binding proteins, Biochemistry

## Abstract

Transcription factor binding to genomic DNA is generally prevented by nucleosome formation, in which the DNA is tightly wrapped around the histone octamer. In contrast, pioneer transcription factors efficiently bind their target DNA sequences within the nucleosome. OCT4 has been identified as a pioneer transcription factor required for stem cell pluripotency. To study the nucleosome binding by OCT4, we prepared human OCT4 as a recombinant protein, and biochemically analyzed its interactions with the nucleosome containing a natural OCT4 target, the *LIN28B* distal enhancer DNA sequence, which contains three potential OCT4 target sequences. By a combination of chemical mapping and cryo-electron microscopy single-particle analysis, we mapped the positions of the three target sequences within the nucleosome. A mutational analysis revealed that OCT4 preferentially binds its target DNA sequence located near the entry/exit site of the nucleosome. Crosslinking mass spectrometry consistently showed that OCT4 binds the nucleosome in the proximity of the histone H3 N-terminal region, which is close to the entry/exit site of the nucleosome. We also found that the linker histone H1 competes with OCT4 for the nucleosome binding. These findings provide important information for understanding the molecular mechanism by which OCT4 binds its target DNA in chromatin.

## Introduction

Transcription factors (TFs) induce the initiation of gene transcription by binding to their target DNA sequences in the genome^1^. In eukaryotes, genomic DNA forms chromatin with the nucleosome as the basic repeating unit, in which a segment containing about 150 base-pairs of DNA is tightly wrapped around a histone octamer^2,3^. The nucleosome formation generally restricts the binding of TFs to their target DNA sequences in the genome. However, a group of TFs, called pioneer TFs, are able to bind to their target DNA sequences within the nucleosome by recruiting downstream transcription factors and chromatin remodelers, and consequently these TFs change the chromatin conformation to regulate the transcription of certain sets of genes^4–6^. The pioneer TF-mediated transcription then regulates cellular differentiation^5^.

Among the pioneer TF family proteins, the overexpression of OCT4, together with SOX2, KLF4, and c-MYC, reportedly promotes the reprogramming of cells to pluripotency^7,8^. At the initial stage of the reprogramming process, OCT4, SOX2, and KLF4 may function as pioneer factors and bind to the “closed chromatin” state, inducing the conversion to the “open chromatin” state^9^. OCT4, SOX2, and KLF4 efficiently bind to reconstituted nucleosomes in vitro, and preferentially accumulate at genomic DNA regions occupied by nucleosomes in vivo^10^.

Human OCT4 is composed of 360 amino acid residues, and two DNA-binding motifs, POU_S_ and POU_HD_, which are located in the middle of the peptide, bind a specific octamer DNA sequence, ATGC(A/T)AAT, and closely related sequences in chromatin^11–13^. A previous crystallographic study revealed that a domain containing the tandem POU_S_ and POU_HD_ of OCT4 binds its target DNA sequence at the major groove^14,15^. In the POU_S_·POU_HD_-DNA complex, the POU_S_ and POU_HD_ motifs sandwich the DNA, and sterically clash with histone binding to the nucleosomal DNA^10^. Therefore, OCT4 may partially peel the DNA from the histone surface upon nucleosome binding. However, the detailed mechanism by which OCT4 specifically recognizes its target DNA sequence in the nucleosome has not been elucidated.

In the present study, we reconstituted the nucleosome with the native *LIN28B* distal enhancer DNA sequence, which contains three potential OCT4 target DNA sequences. The *LIN28B* gene encodes a paralog of the Lin28 protein, Lin28B, which is crucial for reprogramming and pluripotency^16–18^. We found that OCT4 specifically recognizes its target DNA sequence, which is located at the entry/exit site of the nucleosome. A crosslinking mass spectrometry analysis revealed that the OCT4 bound to the nucleosome is located in the proximity of the N-terminal region of histone H3, which is also near the entry/exit site of the nucleosome. Finally, we found that the linker histone H1 competes with OCT4 for nucleosome binding. These new findings provide novel insights toward understanding the molecular mechanism by which OCT4 binds chromatin and regulates the pluripotency of cells.

## Results

### The DNA fragment containing the *LIN28B* distal enhancer region forms a positioned nucleosome in vitro

**Figure 1:**
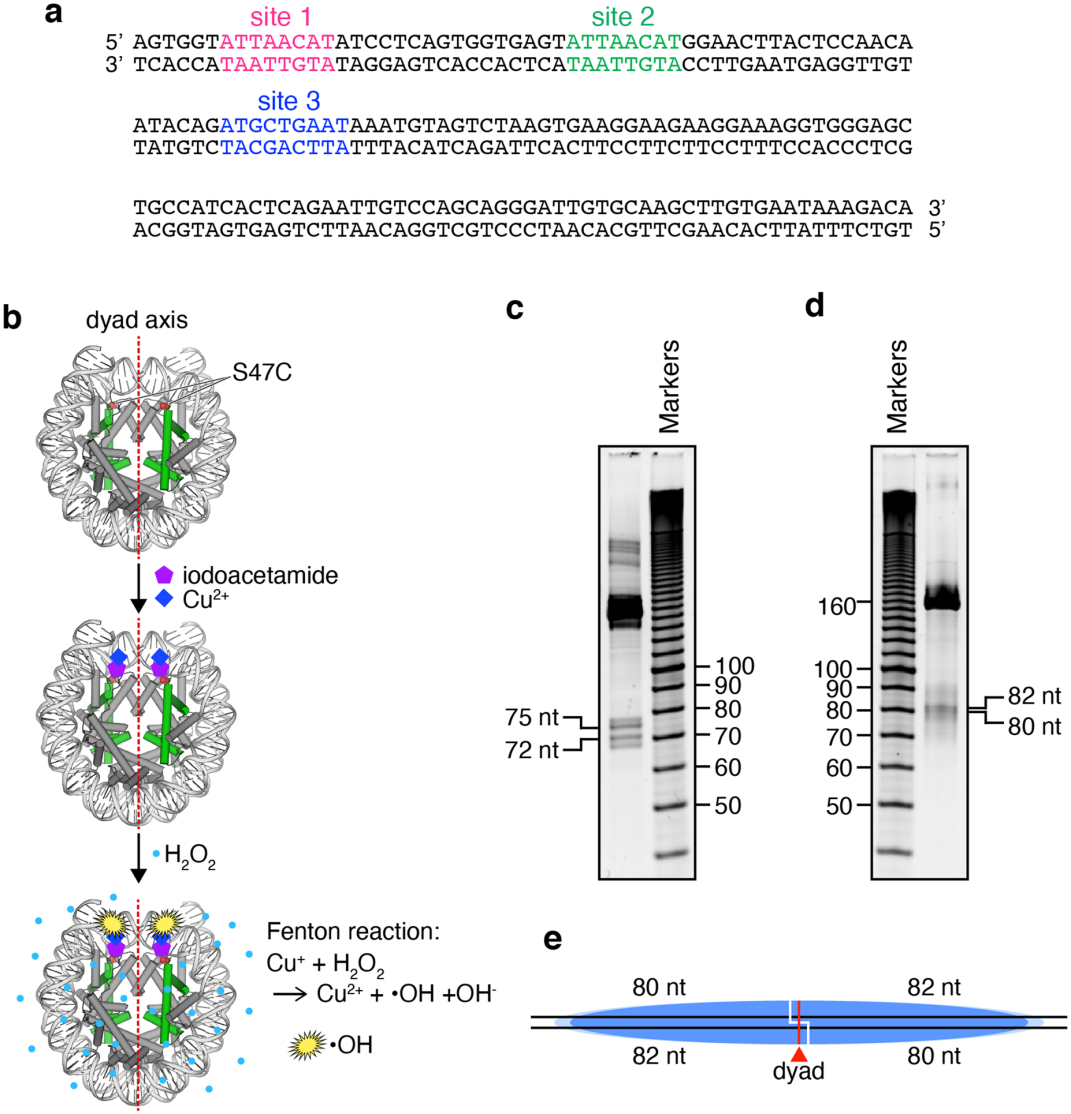
Positioning of the LIN28B nucleosome. (**a**) Sequence of the 162 base-pair *LIN28B* distal enhancer DNA fragment. The three potential OCT4 target sequences are colored magenta (site 1), green (site 2), and blue (site 3). (**b**) Schematic illustration of the chemical probing assay. The nucleosomal DNA is colored light grey. Histone H4 (green) and the other histones (H2A, H2B, H3; colored dark grey) are shown in cylinder representations. The side chains of H4 S47C are represented as red spheres. The nucleosome containing H4 S47C was covalently labeled with iodoacetamide, which anchors the Cu^+^ ion to the DNA backbone near the nucleosomal dyad. The Cu^+^ ion produces hydroxyl radicals (^·^OH) in the presence of hydrogen peroxide (H_2_O_2_), and the DNA backbone near the Cu^+^ ion is cleaved. (**c**,**d**) Chemical probing assay of the nucleosomes containing the 147 base-pair 601 DNA (**c**) and the 162 base-pair LIN28B DNA (**d**). The reaction products were analyzed by denaturing polyacrylamide gel electrophoresis, and were visualized by ethidium bromide staining. (**e**) Positioning of the nucleosome on the 162 base-pair LIN28B DNA fragment. The major position of the nucleosome, defined by 80 and 82 nucleotide (nt) DNA fragments, is represented by a blue ellipse. White lines indicate cleavage sites. The light blue ellipse represents the possible minor positions of the nucleosome. The nucleosomal dyad is represented by a red line.

To test the OCT4 binding to its target DNA sequence in the nucleosome, we reconstituted the nucleosome with a 162 base-pair DNA fragment containing the human *LIN28B* distal enhancer region (LIN28B nucleosome). Three potential OCT4 target sequences (site 1, site 2, and site 3) exist in the 162 base-pair DNA fragment (Fig. 1a). In human fibroblast cells, a positioned nucleosome is reportedly present in the genomic DNA region containing the 162 base-pair DNA segment^10^.

We reconstituted the LIN28B nucleosome by the salt dialysis method in vitro, and tested its nucleosome positioning by the chemical probing assay. To do so, the LIN28B nucleosome was reconstituted with the human canonical histones H2A, H2B, H3.1, and the histone H4 S47C mutant, in which the Ser47 residue was replaced by Cys (Fig. 1b and Fig. S1). In the nucleosome, the H4 Cys residue inserted at position 47 is properly located near the DNA backbones around the nucleosomal dyad, and the DNA strands are chemically cleaved by the Fenton reaction^19,20^ (Fig. 1b). Therefore, the nucleosomal dyad position can be mapped as the cleavage sites by the chemical reaction. A control experiment with the stably positioned nucleosome containing the Widom 601 sequence (147 base pairs) yielded two major 72 and 75 nucleotide bands, confirming that the nucleosomal DNA strands were precisely cleaved around the nucleosomal dyad (Fig. 1c). We then performed the chemical probing assay with the nucleosome containing the human *LIN28B* distal enhancer region (162 base pairs). The LIN28B nucleosome was mainly positioned at the center of the 162 base-pair DNA fragment with linker DNAs on both sides (as revealed by 80- and 82-nt DNA fragments), although the other positions were also observed as smeared bands (Fig. 1d,e). Therefore, the DNA sequence of the human *LIN28B* distal enhancer region (162 base pairs) intrinsically possesses a nucleosome positioning property.

### Cryo-EM structure of the LIN28B nucleosome

**Figure 2:**
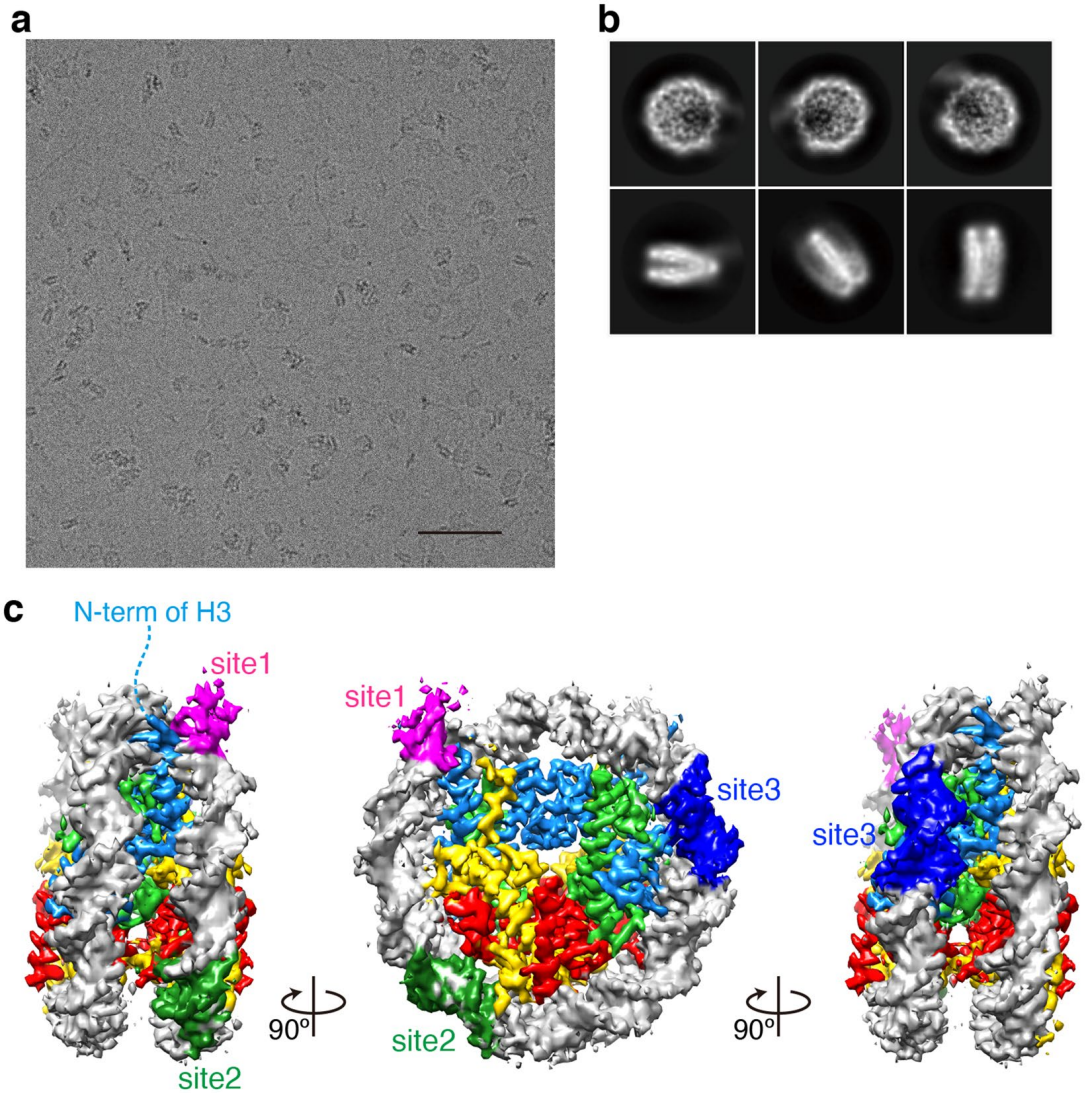
Cryo-EM structure of the LIN28B nucleosome. (**a**) Representative digital micrograph of the LIN28B nucleosome. Scale bar indicates 50 nm. (**b**) Representative 2D class averages of the LIN28B nucleosome, calculated by the RELION software. The box size is 19.8 nm. (**c**) Cryo-EM iso-potential map of the LIN28B nucleosome, contoured at 4.75 sigma above mean density. Histones H2A, H2B, H3, and H4 are colored yellow, red, light blue, and green, respectively. Three OCT4 target DNA sequences are colored magenta, dark green, and blue, respectively. In the left panel, the H3 N-terminal region disordered in the structure is shown by the dashed line.

We then determined the structure of the LIN28B nucleosome by cryo-electron microscopy. The OCT4-LIN28B nucleosome complex was prepared by sucrose gradient, and cryo-EM images were collected (Fig. 2a,b). Since the extra volume corresponding to OCT4 was not obvious probably by the weak or flexible association of OCT4 with the LIN28B nucleosome, the cryo-EM structure of the LIN28B nucleosome without OCT4 was reconstructed, selected, and refined to 3.6 Å resolution (Fig. 2c, Fig. S2, and Table S1). In the structure, the DNA sequence could not be oriented, because the reconstructed nucleosome structure may be a mixture of two opposite orientations. The putative positions of three possible OCT4 binding sites, site 1, site 2, and site 3, are mapped around superhelical location (SHL) 7, SHL4.5, and SHL1.5, respectively, in the LIN28B nucleosome (Fig. 2c).

### OCT4 specifically binds to its target DNA sequence located at the entry/exit site of the nucleosome

**Figure 3:**
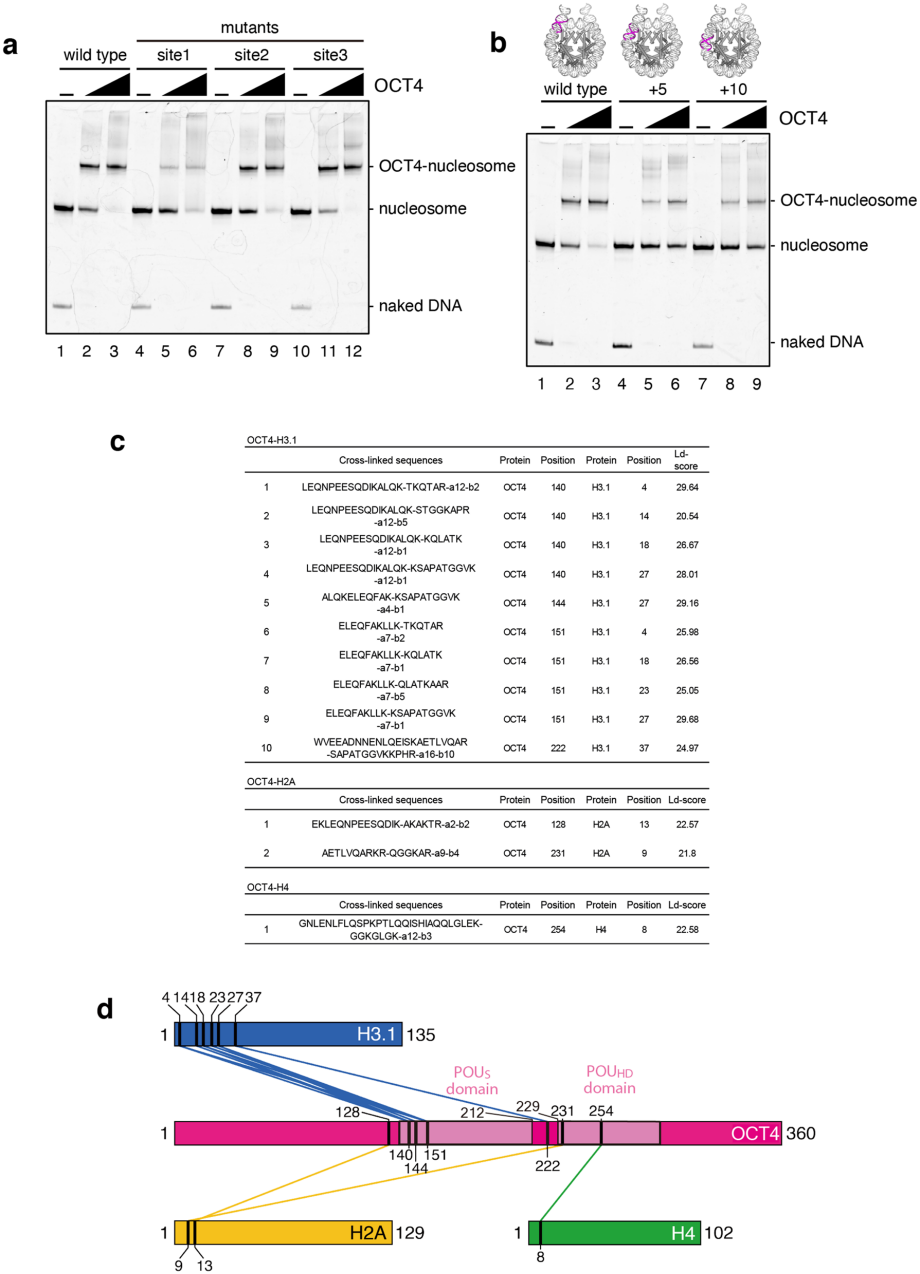
OCT4 binds to the target sequence located near the entry/exit site of the nucleosome. (**a**) Gel-shift assay for the site 1, site 2, and site 3 mutants of the LIN28B nucleosome. The nucleosomes (0.1 µM) were mixed with 0 µM (lanes 1, 4, 7, and 10), 0.1 µM (lanes 2, 5, 8, and 11) and 0.2 µM (lanes 3, 6, 9, and 12) of OCT4, and analyzed by non-denaturing polyacrylamide gel electrophoresis with ethidium bromide staining. Replicated experiments confirmed the reproducibility of the results (Fig. S5a). (**b**) Gel-shift assay using the + 5 and + 10 mutants of the LIN28B nucleosome. The site 1 target (colored magenta) sequence was moved five (+ 5) and ten (+ 10) base pairs toward the nucleosomal dyad (upper illustration). The nucleosomes (0.1 µM) were mixed with 0 µM (lanes 1, 4, and 7), 0.1 µM (lanes 2, 5, and 8) and 0.2 µM (lanes 3, 6, and 9) of OCT4, and analyzed by non-denaturing polyacrylamide gel electrophoresis with ethidium bromide staining. Replicated experiments confirmed the reproducibility of the results (Fig. S5b). (**c**) Peptide sequences identified by XL-MS. The peptide sequences are sorted by their Ld-scores, which are the linear discriminant scores calculated by xQuest/xProphet. The ‘Position’ corresponds to the position of the crosslinked lysine in the protein. (**d**) OCT4-histone interactions in the nucleosome, determined by crosslinking mass spectrometry. OCT4, H2A, H3.1, and H4 are represented by pink, yellow, blue, and green rectangles, respectively. The OCT4 POU_S_·POU_HD_ domains are colored light pink. The interactions between OCT4 and histones (H2A, H3.1, and H4) are shown by lines. Numbers shown above or below the rectangles represent the amino acid residues crosslinked between OCT4 and histones.

To test the OCT4 binding to the LIN28B nucleosome, we purified the His_6_-tagged human OCT4 (5CS) protein (Fig. S3), and performed an electrophoretic mobility shift assay. In the 5CS mutant, all 5 Cys residues in the POU_S_·POU_HD_ domain (C185, C198, C221, C252, and C279) are replaced with Ser to stabilize the protein^21^. Consistent with the previous result^10^, OCT4 efficiently bound to the LIN28B nucleosome, and appeared as a discrete band corresponding to the OCT4-nucleosome complex (Fig. 3a, lanes 1–3). We then mutated one of the three OCT4 target sequences^22^ (site 1–3 mutants), and reconstituted the mutant LIN28B nucleosomes (Fig. S4). Interestingly, OCT4 efficiently formed the specific complexes, when the site 2 and site 3 target sequences were each disrupted (Fig. 3a, lanes 7–12, and Fig. S5a). In contrast, the OCT4 binding was drastically reduced when the site 1 target sequence was disrupted (Fig. 3a, lanes 4–6, and Fig. S5a). The site 1 target sequence is located at the entry/exit site of the LIN28B nucleosome (Fig. 2c). Therefore, these results indicated that OCT4 preferentially binds its target DNA sequence located at the entry/exit site of the nucleosome. Note that the weak, but clear, band corresponding to the specific OCT4-nucleosome complex was observed when site 1 was mutated (Fig. 3a, lanes 4–6, and Fig. S5a). This suggests that OCT4 also binds to site 2 and/or site 3.

We next tested whether the position of the OCT4 target sequence in the nucleosome affects the OCT4 binding. To this end, the site 1 target sequence was moved five (+ 5) and ten (+ 10) base pairs toward the nucleosomal dyad (Fig. 3b and Fig. S4). Since site 2 is located 17 base pairs away from site 1, the + 5 and + 10 sites are positioned between site 1 and site 2 (Fig. S4). As compared to the wild-type LIN28B nucleosome, the OCT4 binding to the LIN28B (+ 5) nucleosome was reduced (Fig. 3b, lanes 1–3 and lanes 4–6, and Fig. S5b). The OCT4-nucleosome binding was also suppressed when the LIN28B (+ 10) nucleosome was used as a template (Fig. 3b, lanes 1–3 and lanes 7–9, and Fig. S5b). In the LIN28B (+ 10) nucleosome, the additional OCT4 binding sequence is supposed to have a similar rotational setting relative to the histone surface with the wild-type LIN28B nucleosome. Therefore, the translational positioning at the entry/exit site of the nucleosome, but not the rotational setting, of the target DNA sequence in the nucleosome may play an important role for efficient OCT4 binding to the nucleosome.

### OCT4 interacts with histone H3 in the nucleosome

We next performed the crosslinking mass spectrometry (XL-MS) analysis, to map the OCT4 binding site relative to the core histones in the nucleosome. The lysine residues located close to the OCT4-nucleosome complex were crosslinked with disuccinimidyl suberate (DSS)-H12/D12. The crosslinked lysine residues between OCT4 and histones were then detected by mass spectrometry (Fig. 3c and Fig. S6). Intriguingly, we found that the POU_S_·POU_HD_ domain of OCT4 was predominantly crosslinked with the N-terminal region of histone H3 (Fig. 3c, and d). In the nucleosome, the N-terminal region of histone H3 is located near the entry/exit DNA sites (Fig. 2c). Therefore, the XL-MS analysis consistently showed that OCT4 predominantly binds to its site 1 target DNA sequence located near the entry/exit site of the nucleosome. It should be noted that a few N-terminal residues of H2A and H4 also crosslinked with OCT4 (Fig. 3c and d). These interactions may be responsible for OCT4 binding to the other nucleosomal regions, such as site 2 and site 3.

### Linker histone H1 competes with OCT4 for nucleosome binding

**Figure 4:**
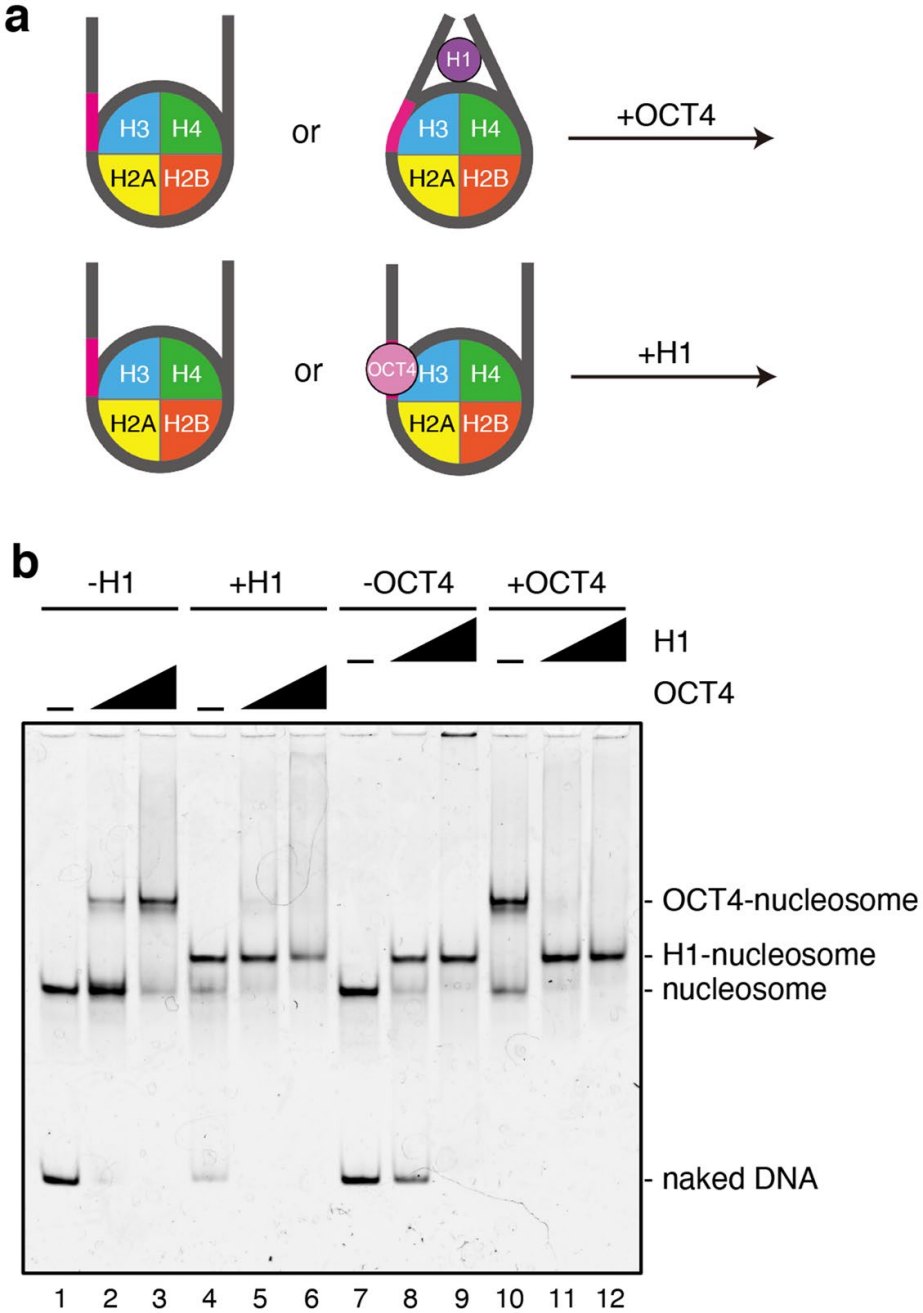
Competitive nucleosome binding assay with linker histone H1 and OCT4. (**a**) Schematic illustration of the competitive nucleosome binding assay. To test OCT4 binding to the H1-nucleosome complex, the nucleosome or the H1.2-nucleosome complex was titrated with OCT4. To test H1 binding to the OCT4-nucleosome complex, the nucleosome or the OCT4-nucleosome complex was titrated with H1.2. (**b**) For the OCT4 titration, the nucleosome (0.1 µM) was preincubated with or without the linker histone H1.2 (0.9 µM), followed by an addition of 0 µM (lanes 1 and 4), 0.15 µM (lanes 2 and 5), and 0.3 µM (lanes 3 and 6) of OCT4. For the H1 titration, the nucleosome (0.1 µM) was preincubated with or without OCT4 (0.3 µM), followed by an addition of 0 µM (lanes 7 and 10), 0.45 µM (lanes 8 and 11), and 0.9 µM (lanes 9 and 12) of H1.2. Samples were analyzed by non-denaturing polyacrylamide gel electrophoresis with ethidium bromide staining. Replicated experiments confirmed the reproducibility of the results (Fig. S8).

Linker histones, such as histones H1 and H5, preferentially bind to the entry/exit DNA regions of the nucleosome^23–26^, and alter the relaxed chromatin conformation to the condensed form^26,27^. In pluripotent cells, the chromatin conformation is largely relaxed, allowing the nucleosome binding of the pioneer transcription factors, including OCT4^28,29^. Interestingly, citrullination of the H1 DNA-binding site reportedly induces H1 displacement and chromatin decondensation in pluripotent cells^30^. The transition of linker histone types also occurs during early embryogenesis^31^. This may play an important role in the global conformational change of chromatin to promote the transcriptionally active somatic state during developmental processes. In these processes, the OCT4 bound to the nucleosome may be replaced by linker histones, suppressing the cells back to the pluripotent state. Therefore, we tested whether the OCT4-nucleosome binding could be replaced by the H1 binding, by performing a competitive nucleosome binding assay (Fig. 4a). In this assay, we used the nucleosome reconstituted with the 193-base-pair 601 sequence DNA, which contains the OCT4 target sequence at its entry/exit site (Fig. S7). We then prepared the H1-nucleosome and OCT4-nucleosome complexes, and performed titration experiments with OCT4 and H1, respectively. When OCT4 was added to the H1-nucleosome complex, neither the OCT4-H1-nucleosome complex formation nor the H1 displacement was observed (Fig. 4b, lanes 1–6, and Fig. S8). In contrast, when H1 was added to the OCT4-nucleosome complex, OCT4 was substantially disassembled from the nucleosome, followed by the formation of the H1-nucleosome complex (Fig. 4b, lanes 7–12, and Fig. S8). These results indicated that the linker histone H1 actually competes with OCT4 for binding to the nucleosome.

## Discussion

In the early stage of cell reprogramming, pioneer TFs bind to their specific target DNA sequences and induce conformational changes of the chromatin architecture. In differentiated cells, the target sites for pioneer TFs are considered to be buried in the closed (condensed) chromatin, which is generally inaccessible to DNA binding proteins. Therefore, pioneer TFs somehow bind their target DNA in chromatin to induce the reprogramming. In the present study, we focused on the pioneer TF, OCT4, and studied its specific binding to the LIN28B nucleosome containing its natural binding DNA sequence, the *LIN28B* distal enhancer DNA sequence.

Previous studies have reported that the *LIN28B* distal enhancer DNA sequence contains target DNA sequences for pioneer TFs, including OCT4, SOX2, and KLF4, which are known to promote induced-pluripotent stem cells (iPSC)^7,8,10^. We performed the OCT4 binding assay with the reconstituted LIN28B nucleosomes containing the OCT4 targeting sequence in various positions. Our chemical probing and cryo-EM analyses revealed that the *LIN28B* distal enhancer DNA fragment forms a positioned nucleosome, and is properly wrapped around the histone octamer (Figs. 1 and 2). We then found that OCT4 stably and preferentially binds its target DNA sequence located at the entry/exit site of the nucleosome (Fig. 3a,b). Our XL-MS data fully supported the favored entry/exit binding of OCT4 in the nucleosome (Fig. 3c). Unexpectedly, OCT4 binding is substantially suppressed when the target DNAs are located in other nucleosomal regions (Fig. 3a,b). Importantly, the suppression of the OCT4 binding may not depend on the rotational setting of the target DNA sequence on the nucleosome surface (Fig. 3a,b). Therefore, OCT4 may efficiently bind its target DNA sequence, if it can be peeled from the histone surface at the entry/exit site of the nucleosome. These findings are consistent with the previous study showing that POU-family TFs prefer the ends of nucleosomal DNA^32^. Recently, the cryo-EM structures of the OCT4-SOX2-nucleosome complexes with designed DNA sequences were reported^33^. In the structures, SOX2 peels the DNA end from the histone surface, and facilitates OCT4 binding to its target site (Fig. S9)^33^. These results, along with our previous findings, suggest that OCT4 prefers to bind the DNA loosely associated with or detached from the histone surface in the nucleosome. In contrast, a single molecule analysis suggested that OCT4 does not discriminate between end-positioned and dyad-positioned target sequences in the nucleosome^34^. This is probably due to the addition of an excess amount of OCT4 relative to the nucleosome, which would favor nonspecific OCT4 binding. It is also possible that OCT4 specifically binds to the internal OCT4 sites of the nucleosome with low affinity, as shown in Fig. 3.

The DNA peeling from the histones could avoid steric clashes between the POU_S_·POU_HD_ domain and core histones. In the previous crystal structures of the POU_S_·POU_HD_-DNA complex, the POU_S_ and POU_HD_ domains bind the DNA from opposite sides and the steric clash with the histones is unavoidable in the nucleosome^10^. Therefore, OCT4 may not efficiently bind the target DNA, if it is located at a region tightly associated with core histones in the nucleosome. In cells, when the OCT4 target DNA sequence is entirely wrapped in the nucleosome, it may not be targeted by OCT4 without repositioning via a nucleosome remodeling mechanism, which may be induced by the other pioneer TFs and nucleosome remodelers. Intriguingly, the cryo-EM OCT4-SOX2-nucleosome structures revealed that OCT4 binds to the nucleosomal target site with assistance from SOX2, which may induce the DNA peeling from the histone surface of the nucleosome^33^. Similar to SOX2, SOX11 also induces the DNA peeling of the nucleosomal DNA ends^35^. Therefore, in the nucleosome, the SOX-family proteins may function to enhance the OCT4 binding by peeling the DNA around the OCT4 targeting sites.

Our XL-MS analysis revealed that OCT4 crosslinks with the H3 N-terminal region, which is located near the DNA entry/exit sites (Fig. 3c,d). Intriguingly, the OCT4-H3 crosslinkings are predominantly observed in the POU_S_ domain, but not in the POU_HD_ domain. This may happen because the POU_S_ domain mainly binds to the nucleosomal DNA at the entry/exit site of the nucleosome. This is consistent with the cryo-EM structure, in which the POU_S_ domain, but not the POU_HD_ domain, of OCT4 binds to the nucleosomal target site^33^. The POU_S_ domain may be a primary recognition module for the nucleosomal target site, and the POU_HD_ domain may have a distinct function in later stages of gene regulation.

We also found that upon nucleosome binding, the linker histone H1 releases the OCT4 bound to the nucleosome (Fig. 4). This is consistent with another pioneer TF, HNF3 (FOXA), which competes with H1 binding on the nucleosome^36^. HNF3 contains a DNA-binding motif with a winged-helix structure, which is similar to that of linker histone H1^37^. In contrast, the POU domain of OCT4 is structurally different from a winged-helix structure^14,15^. Therefore, OCT4 may bind to the nucleosome with a different mode from the winged-helix proteins, such as linker histones and HNF3, and may be evicted from nucleosomes when the winged-helix types of linker DNA-binding proteins are produced in cells.

The OCT4 removal by somatic types of linker histones may function in cellular differentiation. In the early stage of the developmental process, the somatic types of linker histones are expressed at low levels in cells^38,39^. However, the levels of the somatic types of H1s apparently increase progressively upon differentiation^38^. In ES cells, H1 is reportedly more loosely bound to chromatin than in differentiated cells^28,30^. Under these conditions, OCT4 may form a complex with nucleosomes, if the OCT4 target DNA sequences are properly positioned in the nucleosome. This OCT4 binding may accompany the developmental stages of cells. The strong binding of somatic linker histones to chromatin may compete with the OCT4 binding to chromatin, and may contribute to the differentiation of cells to specific types. Consistent with this idea, the depletion of H1 subtypes impairs the differentiation of ES cells^38^.

On the other hand, during the reprogramming process, since OCT4 may not bind to the nucleosome complexed with a linker histone, the linker histone must be evicted from the chromatin before OCT4 can bind to its target DNA. The H1 bound to chromatin reportedly exchanges rapidly in vitro and in vivo^40,41^. This process may be stimulated by histone chaperones, such as NAP1, and modifications of linker histones, which can remove or destabilize the linker histone bound to the nucleosome^30,42^. Further studies are required to clarify the mechanism by which OCT4 modulates the chromatin conformation with linker histones and regulates cell fates.

## Materials and methods

### Preparation of DNA fragments

The 162 base-pair *LIN28B* distal enhancer DNA fragment^10^ (the sequence is AGTGGTATTAACATATCCTCAGTGGTGAGTATTAACATGGAACTTACTCCAACAATACAGATGCTGAATAAATGTAGTCTAAGTGAAGGAAGAAGGAAAGGTGGGAGCTGCCATCACTCAGAATTGTCCAGCAGGGATTGTGCAAGCTTGTGAATAAAGACA) and the 193 base-pair 601 DNA fragment with the OCT4 target sequence (the sequence is ATCGGACCCTATCGCGAGCCAATTAACATAATCCGGTGCCGAGGCCGCTCAATTGGTCGTAGACAGCTCTAGCACCGCTTAAACGCACGTACGCGCTGTCCCCCGCGTTTTAACCGCCAAGGGGATTACTCCCTAGTCTCCAGGCACGTGTCAGATCGGATTCTCCAGGCCTTGTGTCGCGAAGCGCAGCGAT; the introduced OCT4 target sequence is underlined) were each cloned into the pGEM-T-Easy vector (Promega), and used as the templates for PCR amplification. Mutations were introduced by the QuikChange system (Stratagene). The DNA fragments were amplified by PCR and purified by non-denaturing polyacrylamide gel electrophoresis, using a Prep Cell apparatus (Bio-Rad). The eluted DNA fragments were collected in 10 mM Tris–HCl (pH 8.0) buffer containing 0.1 mM EDTA, and were concentrated with a Millipore centrifugal filter.

### Purification of OCT4

Human OCT4 (5CS) was expressed from the pET15b vector (Novagen), in which the thrombin protease recognition site was replaced with the PreScission site. The His_6_-tagged OCT4 (5CS) was produced in the *E. coli* strain BL21-CodonPlus(DE3) RIL, by incubating the culture at 18 °C for 20–24 h after the addition of IPTG to a final concentration of 0.5 mM. The cells were harvested and resuspended in buffer A [50 mM Tris–HCl (pH 7.5), 0.5 M NaCl, 5% glycerol, 7 mM 2-mercaptoethanol, and 1 mM PMSF]. The cells were sonicated on ice, and the lysate was centrifuged at 39,000*g* for 20 min at 4 °C. The pellets were resuspended in buffer B [50 mM Tris–HCl (pH 7.5), 0.5 M NaCl, 5% glycerol, and 7 M guanidine hydrochloride], and incubated overnight at 4 °C. The supernatant containing His_6_-OCT4 (5CS) was mixed with Ni–NTA agarose resin (Qiagen), and the sample was rotated for 1 h at 4 °C. Afterwards, the resin was washed sequentially with buffer C [50 mM Tris–HCl (pH 8.0), 0.5 M NaCl, 6 M guanidine hydrochloride, 5% glycerol, and 10 mM imidazole] and buffer C containing 25 mM imidazole, and the OCT4 protein was eluted with buffer C containing 150 mM imidazole. The eluted sample was concentrated with a Millipore centrifugal filter, and the OCT4 protein was refolded by dripping the sample into × 100 volume of buffer D [30 mM Tris–HCl (pH 7.5), 0.5 M NaCl, and 2 mM 2-mercaptoethanol]. The OCT4 protein was finally purified by Superdex200 (GE Healthcare) gel filtration column chromatography in buffer E [30 mM HEPES–NaOH (pH 7.5), 0.5 M NaCl, and 2 mM 2-mercaptoethanol]. The purified OCT4 protein was concentrated using a Millipore centrifugal filter, and flash frozen in liquid nitrogen.

### Purification of histones and histone complexes

The human histones H2A, H2B, H3.1, and H4 were purified as recombinant proteins, as described previously^43^. Using the purified, lyophilized histones, the H2A-H2B and H3-H4 complexes were reconstituted and isolated^43^. The complexes were flash frozen in liquid nitrogen, and stored at − 80 °C.

### Reconstitution and purification of nucleosomes

The nucleosomes were prepared as described previously^43^. Briefly, a DNA fragment was mixed with the H2A-H2B and H3-H4 complexes in high-salt buffer, and the nucleosomes were reconstituted by the salt dialysis method^43^. To prepare the nucleosome for the chemical probing assay, H4 S47C, in which the Ser47 residue of H4 was replaced by Cys, was used instead of wild-type H4. The resulting nucleosomes were further purified by non-denaturing polyacrylamide gel electrophoresis, using a Prep Cell apparatus (Bio-Rad). The nucleosomes were collected in buffer F [20 mM Tris–HCl (pH 7.5) and 1 mM DTT], and were concentrated using a Millipore centrifugal filter. The nucleosome samples were stored at 4 °C.

### Cryo-electron microscopy

The OCT4-LIN28B nucleosome complex for the cryo-EM analysis was purified by sucrose gradient ultracentrifugation. A gradient was formed with buffer G [10 mM HEPES–NaOH (pH 7.5), 20 mM NaCl, 1 mM DTT, and 5% sucrose] and buffer G containing 15% sucrose, using a gradient maker. For complex formation, the LIN28B nucleosome (0.96 µM) was mixed with OCT4 (nucleosome:OCT4 = 1:6 molar ratio) in buffer H [10 mM Tris–HCl (pH 7.5), 20 mM NaCl, and 1 mM DTT], and was incubated for 1 h on ice. The sample was applied on the top of a gradient, and was centrifuged at 27,000 rpm at 4 °C for 16 h, using a Beckman Sw41Ti rotor. The fractions were analyzed by non-denaturing polyacrylamide gel electrophoresis, and the peak fractions were dialyzed against buffer F. The sample was then concentrated using a Millipore centrifugal filter. Tween-20 was added to a final concentration of 0.00074%, and a 2 µl portion of the sample (0.6 mg/ml) was applied to a glow-discharged Quantifoil holey carbon grid (R1.2/1.3 200-mesh Cu). The grids were blotted for 6 s under 100% relative humidity at 16 °C, and were immediately plunged into liquid ethane, using a Vitrobot Mark IV (Thermo Fisher). Cryo-EM images were collected by the EPU auto acquisition software on a Talos Arctica cryo-electron microscope (Thermo Fisher), operated at 200 kV at a nominal magnification of × 100,000, which renders a pixel size of 1.32 Å at the object scale. Images were recorded under low-dose conditions with 10-s exposure times, using a K2 Summit direct electron detector and a GIF Quantum energy filter (slit width 20 eV) (Gatan) in the counting mode, retaining a total of 40 frames with a total dose of ~ 50 electrons per Å^2^.

### Image processing

In total, 1,877 movies of the LIN28B nucleosome were aligned and integrated using MOTIONCOR2 (https://emcore.ucsf.edu/ucsf-motioncor2)^44^, with dose weighting. The contrast transfer function (CTF) was estimated by CTFFIND4 (https://grigoriefflab.janelia.org/ctf)^45^ from the digital micrographs, with dose weighting. In total, 1,274 images were selected based on the CTF fit correlation to approximately 5 Å resolution. RELION 3.0 (https://www2.mrc-lmb.cam.ac.uk/relion/index.php/Main_Page)^46^ was used for all subsequent image processing operations. Subsequently, 325,272 particles of the LIN28B nucleosome were picked automatically, with a box-size of 150 × 150 pixels. Two-dimensional classification to remove bad particles resulted in the selection of 264,297 particles. The crystal structure of a canonical nucleosome (PDB: 3LZ0) in the low-pass filtered to 60 Å was used as the initial three-dimensional reference. The best classes containing 150,721 particles, in which the nucleosomal DNA was fully wrapped around the histone octamer, were selected from the three-dimensional classification. The three-dimensional refinement of the LIN28B nucleosome was performed, followed by particle polishing and two rounds of CTF refinement. The final three-dimensional map of the LIN28B nucleosome was sharpened with an exponential B-factor (− 50.7 Å^2^). The gold standard Fourier Shell Correlation (FSC) at the FSC = 0.143 criterion^47^ was used for the resolution estimation of the refined map. The local resolution map of the LIN28B nucleosome was calculated with ResMap (https://resmap.sourceforge.net)^48^. The final three-dimensional map was normalized with MAPMAN (https://xray.bmc.uu.se/usf/mapman_man.html)^49^, and the iso-electron potential surface of the LIN28B nucleosome was visualized with UCSF Chimera (https://www.cgl.ucsf.edu/chimera/)^50^.

### Analysis of nucleosome positioning by chemical probing assay

The in vitro chemical probing assay was performed according to the previously reported method^43^. The LIN28B nucleosome containing H4 S47C (37.4 pmol) was labeled with N-(1,10-phenanthrolin-5-yl) iodoacetamide at a molar ratio of 1:50 (nucleosome:iodoacetamide) in buffer I [10 mM Tris–HCl (pH 7.5) and 50 mM NaCl] overnight at 4 °C. To remove the excess label, the sample solution was exchanged with buffer J [50 mM Tris–HCl (pH 7.5) and 2.5 mM NaCl], using a Millipore centrifugal filter. CuCl_2_ was added to a final concentration of 0.15 mM, and the sample was incubated for 2 min at room temperature. The sample solution was then exchanged with buffer J, using a Millipore centrifugal filter. To initiate the reaction, MPA (3-mercaptopropionic acid) and H_2_O_2_ were added to a final concentration of 6 mM each, and the sample was incubated for 5 min at room temperature. To stop the reaction, neocuproine was added to a final concentration of 2.8 mM. The DNA fragments were then extracted with phenol–chloroform, and precipitated with ethanol. The purified DNA fragments were resuspended in Hi-Di Formamide, and incubated at 95 °C for 5 min. The DNA samples were analyzed by electrophoresis on a denaturing 10% polyacrylamide gel containing 7 M urea. The DNA bands were visualized by ethidium bromide staining.

### Gel mobility shift assay

Each reaction was performed in a total volume of 10 µl. The LIN28B nucleosome (0.1 µM) was mixed with 0 µM, 0.1 µM, or 0.2 µM of His_6_-OCT4 (5CS). The samples were incubated for 1 h on ice, in a solution containing 2 mM Tris–HCl (pH 7.5), 13 mM HEPES–NaOH (pH 7.5), 50 mM NaCl, 0.1 mM DTT, 2.2 mM β-mercaptoethanol, and 0.03% NP-40. After the incubation, the reaction products were analyzed by non-denaturing polyacrylamide gel electrophoresis with ethidium bromide staining.

### Competitive nucleosome binding assay with a linker histone H1 and OCT4

Each reaction was performed in a total volume of 10 µl. For the OCT4 titration experiments, the 193 base-pair 601 nucleosome containing the OCT4 target sequence (final 0.1 µM) was preincubated with or without the linker histone H1.2 (final 0.9 µM) for 20 min on ice. OCT4 (final 0 µM, 0.15 µM, and 0.3 µM) was then added and the mixture was further incubated for 1 h on ice. For the H1 titration experiments, the 193 base-pair 601 nucleosome containing the OCT4 target sequence (final 0.1 µM) was preincubated with or without OCT4 (final 0.3 µM). H1.2 (final 0 µM, 0.45 µM, and 0.9 µM) was then added and the mixture was further incubated for 1 h on ice. The final reaction solution contained 30 mM Tris–HCl (pH 7.5), 1.5 mM HEPES–NaOH (pH 7.5), 65 mM NaCl, 0.1 mM DTT, 2.9 mM β-mercaptoethanol, 0.001% PGA, and 4% glycerol. The reaction mixtures were analyzed by non-denaturing polyacrylamide gel electrophoresis with ethidium bromide staining.

### Crosslinking mass spectrometry

Crosslinking mass spectrometry was performed as described previously^51,52^. The LIN28B nucleosome (4.3 μM) was mixed with OCT4 in a nucleosome:OCT4 = 1:4 molar ratio, and was incubated on ice for 1 h. The sample was then crosslinked with 9.6 mM DSS-H12/D12 (Creative Molecules) at 30 °C for 30 min. The reaction was stopped by adding 48 mM ammonium bicarbonate, and then incubated at 30 °C for 15 min. The sample was reduced, alkylated, and then digested by sequencing-grade endopeptidase Trypsin/Lys-C Mix (Promega), at an enzyme–substrate ratio of 1:50 wt/wt. The digested sample was applied to a Superdex 30 Increase 3.2/300 (GE Healthcare) column, using buffer containing 25% acetonitrile and 0.1% TFA. The eluted fractions (150 μl) were collected, dried, and re-dissolved in 0.1% TFA. The samples were analyzed by liquid chromatography tandem mass spectrometry (LC/MS–MS), using an LTQ-Orbitrap Velos mass spectrometer (Thermo Fisher Scientific) equipped with a Zaplous Advance nano UHPLC HTS-PAL xt System (AMR). The crosslinked peptides were identified using the xQuest/xProphet software (https://proteomics.ethz.ch/)^51^, and the crosslinks were visualized using the webserver xVis (https://xvis.genzentrum.lmu.de/login.php)^53^. The mass spectrometry raw data used in this study have been deposited to the proteomeXchange Consortium via the JPOST repository (PXD019160, https://proteomecentral.proteomexchange.org/cgi/GetDataset?ID=PXD019160)^54^.

## Supplementary information

Supplementary Information.

## Data Availability

The cryo-EM map of the LIN28B nucleosome has been deposited in the Electron Microscopy Data Bank, with the EMDB ID codes EMD-30070. The mass spectrometry raw data have been deposited to the proteomeXchange Consortium via the JPOST repository, with the ID code PXD019160.

## References

[1] Lambert SA (2018). The human transcription factors. Cell.

[2] Luger K, Mäder AW, Richmond RK, Sargent DF, Richmond TJ (1997). Crystal structure of the nucleosome core particle at 2.8 Å resolution. Nature.

[3] Koyama M, Kurumizaka H (2018). Structural diversity of the nucleosome. J. Biochem..

[4] Iwafuchi-Doi M, Zaret KS (2014). Pioneer transcription factors in cell reprogramming. Genes Dev..

[5] Zaret KS, Mango SE (2016). Pioneer transcription factors, chromatin dynamics, and cell fate control. Curr. Opin. Genet. Dev..

[6] Makowski MM, Gaullier G, Luger K (2020). Picking a nucleosome lock: sequence- and structure-specific recognition of the nucleosome. J. Biosci..

[7] Takahashi K, Yamanaka S (2006). Induction of pluripotent stem cells from mouse embryonic and adult fibroblast cultures by defined factors. Cell.

[8] Takahashi K (2007). Induction of pluripotent stem cells from adult human fibroblasts by defined factors. Cell.

[9] Soufi A, Donahue G, Zaret KS (2012). Facilitators and impediments of the pluripotency reprogramming factors’ initial engagement with the genome. Cell.

[10] Soufi A (2015). Pioneer transcription factors target partial DNA motifs on nucleosomes to initiate reprogramming. Cell.

[11] Schöler HR, Hatzopoulos AK, Balling R, Suzuki N, Gruss P (1989). A family of octamer-specific proteins present during mouse embryogenesis: evidence for germline-specific expression of an Oct factor. EMBO J..

[12] Rosner MH (1990). A POU-domain transcription factor in early stem cells and germ cells of the mammalian embryo. Nature.

[13] Verrijzer CP (1992). The DNA binding specificity of the bipartite POU domain and its subdomains. EMBO J..

[14] Reményi A (2003). Crystal structure of a POU/HMG/DNA ternary complex suggests differential assembly of Oct4 and Sox2 on two enhancers. Genes Dev..

[15] Esch D (2013). A unique Oct4 interface is crucial for reprogramming to pluripotency. Nat. Cell Biol..

[16] Yu J (2007). Induced pluripotent stem cell lines derived from human somatic cells. Science.

[17] Hanna J (2009). Direct cell reprogramming is a stochastic process amenable to acceleration. Nature.

[18] Tsialikas J, Romer-Seibert J (2015). LIN28: roles and regulation in development and beyond. Development.

[19] Brogaard KR, Xi L, Wang JP, Widom J (2012). A chemical approach to mapping nucleosomes at base pair resolution in yeast. Methods Enzymol..

[20] Flaus A, Luger K, Tan S, Richmond TJ (1996). Mapping nucleosome position at single base-pair resolution by using site-directed hydroxyl radicals. Proc. Natl. Acad. Sci. U S A.

[21] Reményi A, Pohl E, Schöler HR, Wilmanns M (2001). Crystallization of redox-insensitive Oct1 POU domain with different DNA-response elements. Acta Crystallogr. D Biol. Crystallogr..

[22] Smith AEF, Ford KG (2005). Use of altered-specificity binding Oct-4 suggests an absence of pluripotent cell-specific cofactor usage. Nucleic Acids Res..

[23] Zhou BR (2013). Structural insights into the histone H1-nucleosome complex. Proc. Natl. Acad. Sci. U S A..

[24] Zhou BR (2015). Structural mechanisms of nucleosome recognition by linker histones. Mol. Cell.

[25] Bednar J (2017). Structure and dynamics of a 197 bp nucleosome in complex with linker histone H1. Mol. Cell.

[26] Garcia-Saez I (2018). Structure of an H1-bound 6-nucleosome array reveals an untwisted two-start chromatin fiber conformation. Mol. Cell.

[27] Song F (2014). Cryo-EM study of the chromatin fiber reveals a double helix twisted by tetranucleosomal units. Science.

[28] Meshorer E (2006). Hyperdynamic plasticity of chromatin proteins in pluripotent embryonic stem cells. Dev. Cell.

[29] Gaspar-Maia A, Alajem A, Meshorer E, Ramalho-Santos M (2014). Open chromatin in pluripotency and reprogramming. Nat. Rev. Mol. Cell Biol..

[30] Christophorou MA (2014). Citrullination regulates pluripotency and histone H1 binding to chromatin. Nature.

[31] Dimitrov S, Almouzni G, Dasso M, Wolffe AP (1993). Chromatin transitions during early Xenopus embryogenesis: changes in histone H4 acetylation and in linker histone type. Dev. Biol..

[32] Zhu F (2018). The interaction landscape between transcription factors and the nucleosome. Nature.

[33] Michael AK (2020). Mechanisms of OCT4-SOX2 motif readout on nucleosomes. Science.

[34] Li S, Zheng EB, Zhao L, Liu S (2019). Nonreciprocal and conditional cooperativity directs the pioneer activity of pluripotency transcription factors. Cell Rep..

[35] Dodonova SO (2020). Nucleosome-bound SOX2 and SOX11 structures elucidate pioneer factor function. Nature.

[36] Cirillo LA (1998). Binding of the winged-helix transcription factor HNF3 to a linker histone site on the nucleosome. EMBO J..

[37] Clark K, Halay E, Lai E, Burley SK (1993). Co-crystal structure of the HNF-3/fork head DNA-recognition motif resembles histone H5. Nature.

[38] Zhang Y (2012). Histone H1 depletion impairs embryonic stem cell differentiation. PLoS Genet..

[39] Izzo A (2017). Dynamic changes in H1 subtype composition during epigenetic reprogramming. J. Cell Biol..

[40] Caron F, Thomas JO (1981). Exchange of histone H1 between segments of chromatin. J. Mol. Biol..

[41] Misteli T, Gunjan A, Hock R, Bustin M, Brown DT (2000). Dynamic binding of histone H1 to chromatin in living cells. Nature.

[42] Machida S (2015). Nap1 stimulates homologous recombination by RAD51 and RAD54 in higher-ordered chromatin containing histone H1. Sci. Rep..

[43] Kujirai T (2018). Methods for preparing nucleosomes containing histone variants. Methods Mol. Biol..

[44] Zheng SQ (2017). MotionCor2: anisotropic correction of beam-induced motion for improved cryo-electron microscopy. Nat. Methods.

[45] Rohou A, Grigorieff N (2015). CTFFIND4: fast and accurate defocus estimation from electron micrographs. J. Struct. Biol..

[46] Zivanov J (2018). New tools for automated high-resolution cryo-EM structure determination in RELION-3. Elife.

[47] Scheres SH (2016). Processing of structurally heterogeneous cryo-EM data in RELION. Methods Enzymol..

[48] Kucukelbir A, Sigworth FJ, Tagare HD (2004). Quantifying the local resolution of cryo-EM density maps. Nat. Methods.

[49] Kleywegt GJ (2004). The uppsala electron-density server. Acta Crystallogr. D Biol. Crystallogr..

[50] Pettersen EF (2004). UCSF Chimera—A visualization system for exploratory research and analysis. J. Comput. Chem..

[51] Leitner A, Walzthoeni T, Aebersold R (2014). Lysine-specific chemical cross-linking of protein complexes and identification of cross-linking sites using LC-MS/MS and the xQuest/xProphet software pipeline. Nat. Protoc..

[52] Kobayashi W (2019). Structural and biochemical analyses of the nuclear pore complex component ELYS identify residues responsible for nucleosome binding. Commun. Biol..

[53] Grimm M, Zimniak T, Kahraman A, Herzog F (2015). xVis: a web server for the schematic visualization and interpretation of crosslink-derived spatial restraints. Nucleic Acids Res..

[54] Okuda S, Watanabe Y, Moriya Y, Kawano S, Yamamoto T, Matsumoto M, Takami T, Kobayashi D, Araki N, Yoshizawa CA, Tabata T, Sugiyama N, Goto S, Ishihama Y (2017). jPOSTrepo: an international standard data repository for proteomes. Nucleic Acids Res..

