# Supplementary material for: Nucleosome binding by the pioneer transcription factor OCT4
Source: Sci Rep. 2020 Jul 16;10:11832. doi: 10.1038/s41598-020-68850-1 (PMC7367260; doi:10.1038/s41598-020-68850-1)
Supplement: Supplementary file 1 — Supplementary Information. [file 41598_2020_68850_MOESM1_ESM.pdf]

Supplemental Information

Nucleosome binding by the pioneer transcription factor OCT4

Kenta Echigoya, Masako Koyama, Lumi Negishi, Yoshimasa Takizawa, Yuka Mizukami, Hideki Shimabayashi, Akari Kuroda, and Hitoshi Kurumizaka

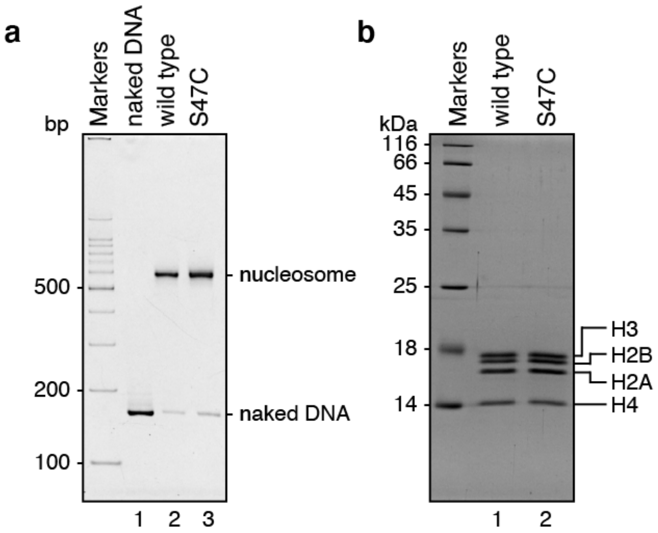

Supplementary Figure S1. Purification of the LIN28B nucleosome for chemical probing assay. The purified LIN28B nucleosomes containing wild-type H4 (control) or the H4 S47C mutant were analyzed by non-denaturing polyacrylamide gel electrophoresis with EtBr staining **(a)**, and SDS-polyacrylamide gel electrophoresis with Coomassie Brilliant Blue staining **(b)**.

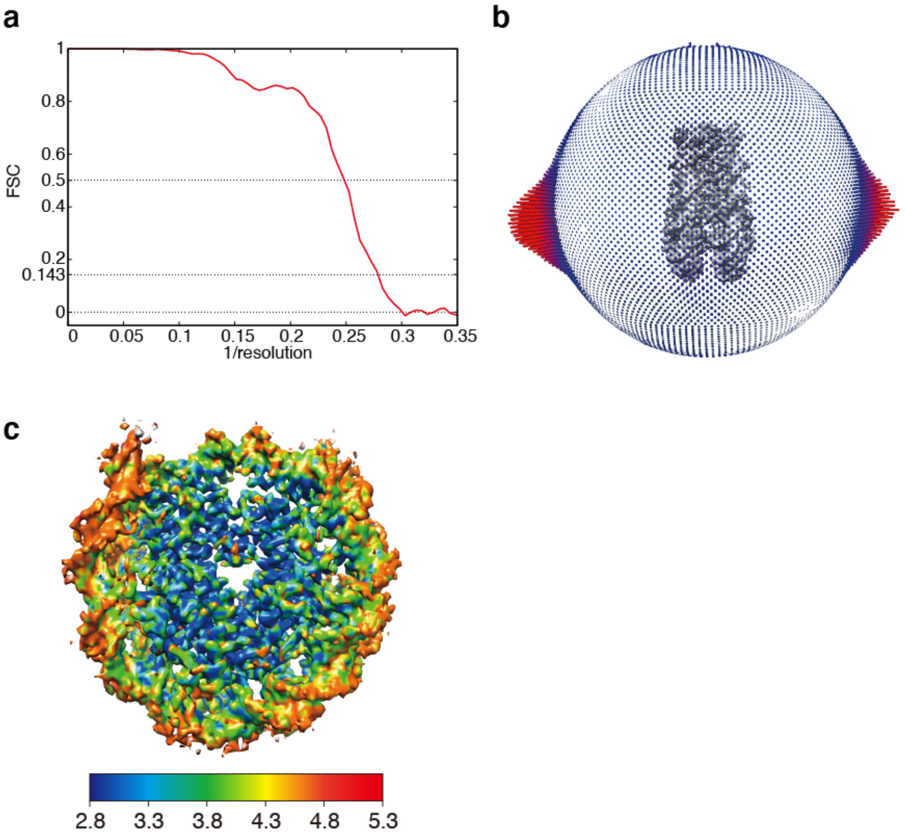

Supplementary Figure S2. Cryo-EM analysis of the LIN28B nucleosome. (a) Fourier Shell Correlation (FSC) calculated between independently refined image datasets of the LIN28B nucleosome. The overall resolution of the LIN28B nucleosome is 3.6 Å (FSC=0.143). **(b)** Euler angle distribution map of all particles contributing to the 3D reconstruction of the LIN28B nucleosome. **(c)** Local resolution map of the LIN28B nucleosome, showing the resolution range between 2.8 to 5.3 Å.

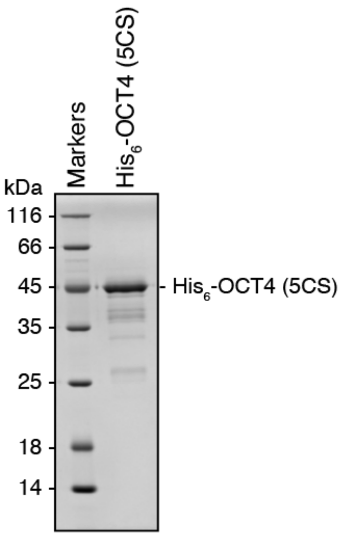

Supplementary Figure S3. Purification of human OCT4. The purified His$_6$-tagged human OCT4 (5CS) protein was analyzed by SDS-polyacrylamide gel electrophoresis with Coomassie Brilliant Blue staining.

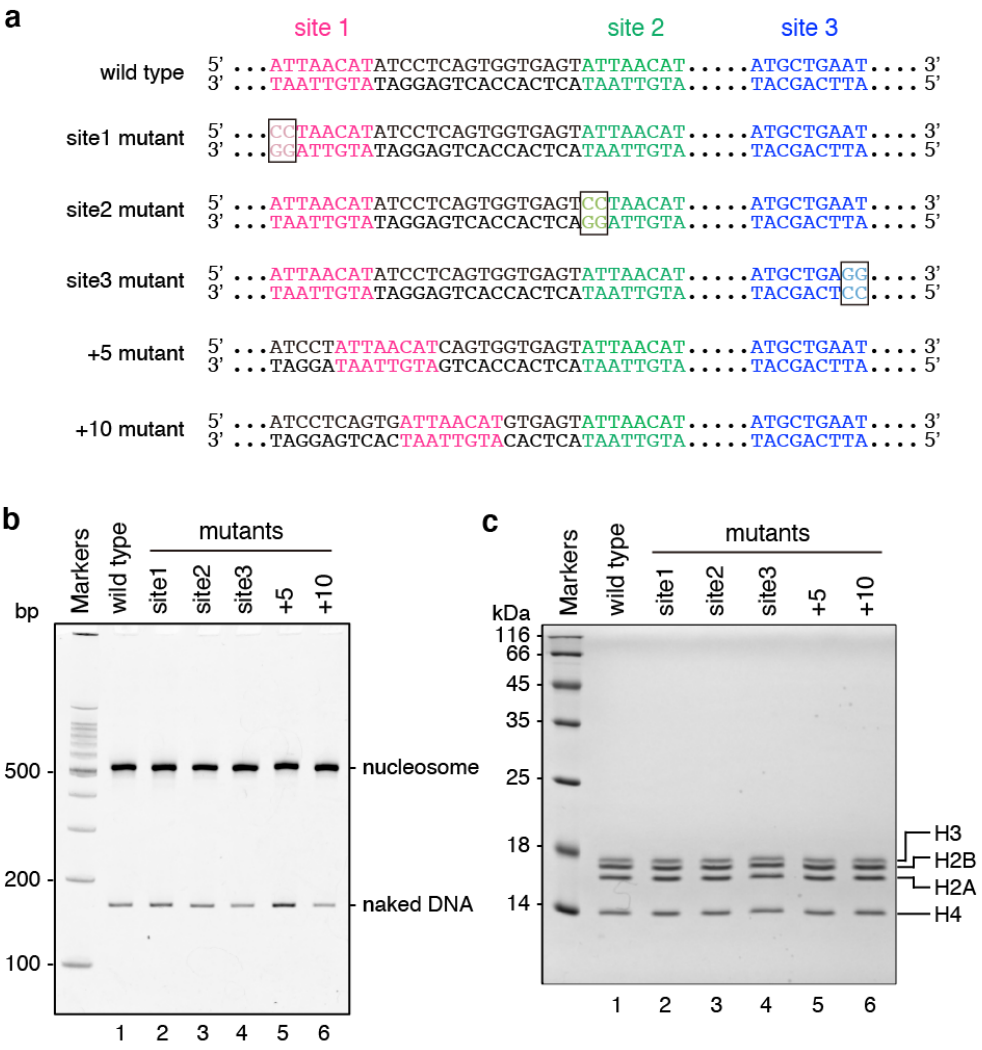

Supplementary Figure S4. Purification of the mutant LIN28B nucleosomes. (a) Design of mutations in the 162 base-pair LIN28B DNA. Site 1, site 2, and site 3 are colored magenta, green, and blue, respectively. For the site 1, site 2, and site 3 mutants, the mutated bases are colored light pink, light green, and light blue with black squares, respectively. **(b, c)** Purification of the mutant LIN28B nucleosomes reconstituted with the mutant LIN28B DNAs shown in **(a)**. The purified nucleosomes were analyzed by non-denaturing polyacrylamide gel electrophoresis with EtBr staining **(b)** and SDS-polyacrylamide gel electrophoresis with Coomassie Brilliant Blue staining **(c)**.

a

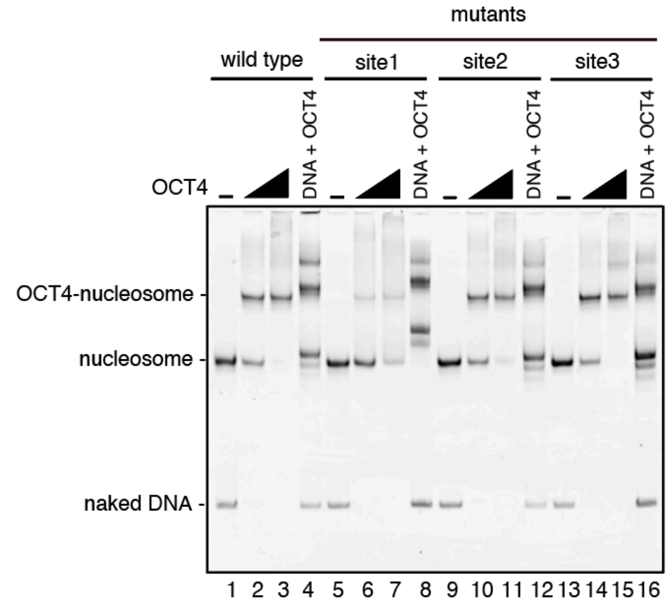

b

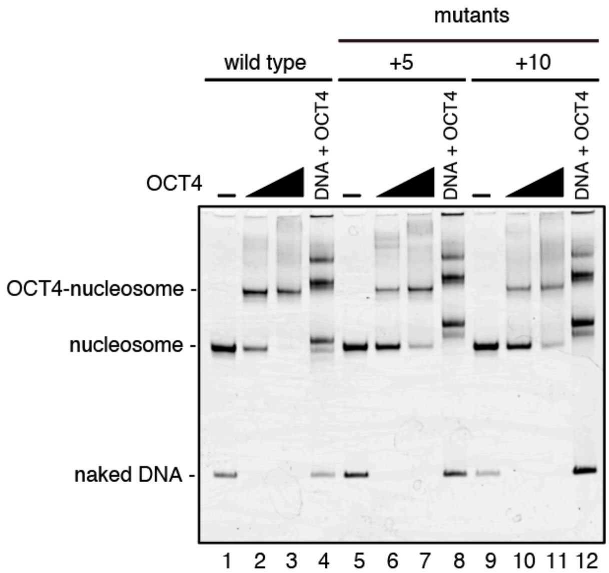

Supplementary Figure S5. Replicated experiments for Fig. 3a and 3b. (a) Gel-shift assay for the site 1, site 2, and site 3 mutants of the LIN28B nucleosome or naked DNA with the *LIN28B* distal enhancer sequence. The nucleosomes were titrated with OCT4, and analyzed by non-denaturing polyacrylamide gel electrophoresis with ethidium bromide staining. In the experiments with naked DNA (lanes 4, 8, 12, and 16), the DNA and OCT4 concentrations were 0.1 µM and 0.2 µM, respectively. **(b)** Gel-shift assay using the +5 and +10 mutants of the LIN28B nucleosome or DNA. The nucleosomes and DNA were titrated with OCT4, and analyzed by non-denaturing polyacrylamide gel electrophoresis with ethidium bromide staining. In the DNA and OCT4 binding assay, the DNA and OCT4 concentrations were 0.1 and 0.2 µM, respectively.

a

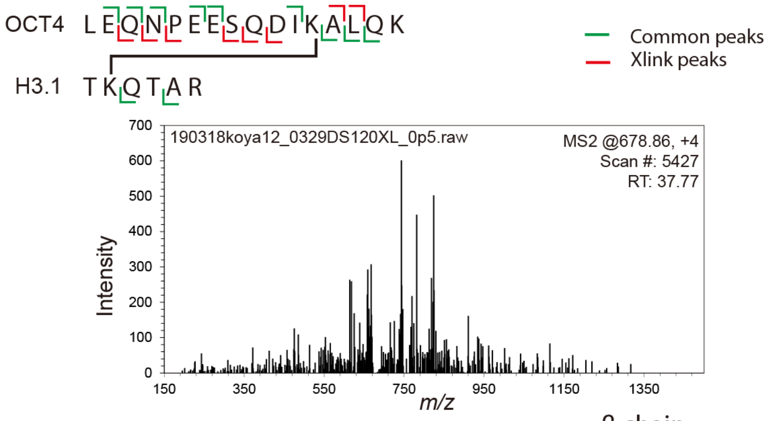

α chain

a common b standard	+1	114.09	243.13	371.19	485.24	582.29	711.33	840.37	927.41	1055.46	1170.49	1283.58	-	-	-	-	-
	+2	57.55	122.07	186.1	243.12	291.65	356.17	420.69	464.21	528.24	585.75	642.29	-	-	-	-	-
	+3	38.7	81.72	124.4	162.42	194.77	237.78	280.8	309.81	352.49	390.84	428.53	-	-	-	-	-
a xlink b standard	+2	-	-	-	-	-	-	-	-	-	-	-	1127.07	1162.59	1219.13	1283.16	1347.21
	+3	-	-	-	-	-	-	-	-	-	-	-	751.72	775.4	813.09	855.78	898.48
	+4	-	-	-	-	-	-	-	-	-	-	-	564.04	581.8	610.07	642.08	674.11
AA		L	E	Q	N	P	E	E	S	Q	D	I	K	A	L	Q	K
a common y standard	+1	-	-	-	-	-	-	-	-	-	-	-	-	459.29	388.26	275.17	147.11
	+2	-	-	-	-	-	-	-	-	-	-	-	-	230.15	194.63	138.09	74.06
	+3	-	-	-	-	-	-	-	-	-	-	-	-	153.77	130.09	92.4	49.71
a xlink y standard	+2	1356.21	1299.67	1235.15	1171.12	1114.1	1065.57	1001.05	936.53	893.02	828.99	771.47	714.93	-	-	-	-
	+3	904.48	866.78	823.77	781.08	743.07	710.72	667.7	624.69	595.68	552.99	514.65	476.96	-	-	-	-
	+4	678.61	650.34	618.08	586.06	557.55	533.29	501.03	468.77	447.01	415	386.24	357.97	-	-	-	-

β chain

b common b standard	+1	102.06	-	-	-	-	-
	+2	51.53	-	-	-	-	-
	+3	34.69	-	-	-	-	-
b xlink b standard	+2	-	1119.09	1183.12	1233.64	1269.16	1347.21
	+3	-	746.39	789.08	822.76	846.44	898.48
	+4	-	560.05	592.06	617.32	635.08	674.11
AA		T	K	Q	T	A	R
b common y standard	+1	-	-	475.26	347.2	246.16	175.12
	+2	-	-	238.14	174.11	123.58	88.06
	+3	-	-	159.09	116.41	82.72	59.05
b xlink y standard	+2	1356.21	1305.69	-	-	-	-
	+3	904.48	870.8	-	-	-	-
	+4	678.61	653.35	-	-	-	-

b

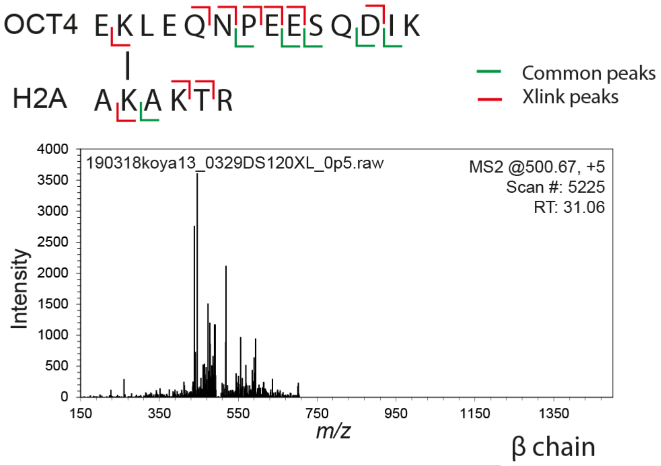

α chain

a common b standard	+1	130.05	-	-	-	-	-	-	-	-	-	-	-	-	-
	+2	65.53	-	-	-	-	-	-	-	-	-	-	-	-	-
	+3	44.02	-	-	-	-	-	-	-	-	-	-	-	-	-
a xlink b standard	+2	-	535.32	591.86	656.39	720.42	777.44	825.96	890.48	955.01	998.52	1062.55	1120.06	1176.61	1240.65
	+3	-	357.22	394.91	437.93	480.61	518.63	550.98	593.99	637.01	666.02	708.7	747.05	784.74	827.44
	+4	-	268.17	296.44	328.7	360.71	389.22	413.49	445.75	478.01	499.76	531.78	560.54	588.81	620.83
	+5	-	214.73	237.35	263.16	288.77	311.58	330.99	356.8	382.61	400.01	425.62	448.63	471.25	496.87
AA		E	K	L	E	Q	N	P	E	E	S	Q	D	I	K
a common y standard	+1	-	-	1429.68	1316.6	1187.55	1059.5	945.45	848.4	719.36	590.31	503.28	375.22	260.2	147.11
	+2	-	-	715.34	658.8	594.28	530.25	473.23	424.7	360.18	295.66	252.15	188.12	130.6	74.06
	+3	-	-	477.23	439.54	396.52	353.84	315.82	283.47	240.46	197.44	168.43	125.75	87.4	49.71
a xlink y standard	+2	1249.66	1185.14	-	-	-	-	-	-	-	-	-	-	-	-
	+3	833.44	790.43	-	-	-	-	-	-	-	-	-	-	-	-
	+4	625.33	593.07	-	-	-	-	-	-	-	-	-	-	-	-
	+5	500.47	474.66	-	-	-	-	-	-	-	-	-	-	-	-

β chain

b common b standard	+1	72.04	-	-	-	-	-
	+2	36.53	-	-	-	-	-
	+3	24.69	-	-	-	-	-
b xlink b standard	+2	-	1012.51	1048.03	1112.08	1162.6	1240.65
	+3	-	675.34	699.02	741.72	775.4	827.44
	+4	-	506.76	524.52	556.54	581.81	620.83
	+5	-	405.61	419.82	445.44	465.65	496.87
AA		A	K	A	K	T	R
b common y standard	+1	-	-	475.3	404.26	276.17	175.12
	+2	-	-	238.15	202.63	138.59	88.06
	+3	-	-	159.1	135.43	92.73	59.05
b xlink y standard	+2	1249.66	1214.14	-	-	-	-
	+3	833.44	809.76	-	-	-	-
	+4	625.33	607.57	-	-	-	-
	+5	500.47	486.26	-	-	-	-

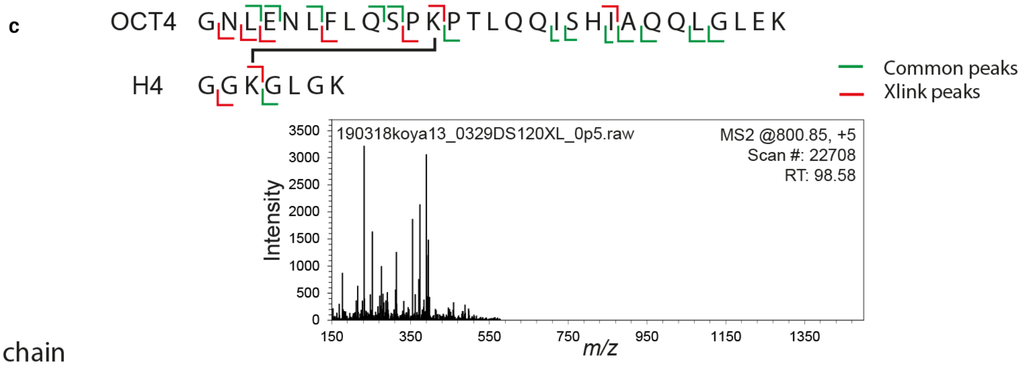

α chain

		G	N	L	E	N	L	F	L	Q	S	P	K
a common b standard	+1	58.03	172.07	285.16	414.2	528.24	641.33	788.39	901.48	1029.54	1116.57	1213.62	-
	+2	29.52	86.54	143.08	207.6	264.62	321.17	394.7	451.24	515.27	558.79	607.31	-
	+3	20.01	58.03	95.72	138.74	176.75	214.45	263.47	301.16	343.85	372.86	405.21	-
a xlink b standard	+2	-	-	-	-	-	-	-	-	-	-	-	1048.08
	+3	-	-	-	-	-	-	-	-	-	-	-	699.06
	+4	-	-	-	-	-	-	-	-	-	-	-	524.54
	+5	-	-	-	-	-	-	-	-	-	-	-	419.84
AA		G	N	L	E	N	L	F	L	Q	S	P	K
a common y standard	+1	-	-	-	-	-	-	-	-	-	-	-	-
	+2	-	-	-	-	-	-	-	-	-	-	-	-
	+3	-	-	-	-	-	-	-	-	-	-	-	-
a xlink y standard	+2	1999.61	1971.1	1914.08	1857.53	1793.01	1735.99	1679.45	1605.91	1549.37	1485.34	1441.83	1393.3
	+3	1333.41	1314.4	1276.39	1238.69	1195.68	1157.66	1119.97	1070.95	1033.25	990.56	961.55	929.2
	+4	1000.31	986.05	957.54	929.27	897.01	868.5	840.23	803.46	775.19	743.18	721.42	697.15
	+5	800.45	789.04	766.23	743.62	717.81	695	672.38	642.97	620.35	594.74	577.34	557.93

		P	T	L	Q	Q	I	S	H	I	A	Q	Q	L	G	L	E	K
a common b standard	+1	-	-	-	-	-	-	-	-	-	-	-	-	-	-	-	-	-
	+2	-	-	-	-	-	-	-	-	-	-	-	-	-	-	-	-	-
	+3	-	-	-	-	-	-	-	-	-	-	-	-	-	-	-	-	-
a xlink b standard	+2	1096.61	1147.13	1203.67	1267.7	1331.73	1388.27	1431.79	1500.32	1556.86	1592.38	1656.41	1720.44	1776.98	1805.49	1862.03	1926.56	1990.6
	+3	731.41	765.09	802.79	845.47	888.16	925.85	954.86	1000.55	1038.24	1061.92	1104.61	1147.3	1184.99	1204	1241.69	1284.71	1327.4
	+4	548.81	574.07	602.34	634.36	666.37	694.64	716.4	750.66	778.93	796.69	828.71	860.72	888.99	903.25	931.52	963.78	995.81
	+5	439.25	459.46	482.07	507.69	533.3	555.91	573.32	600.73	623.35	637.56	663.17	688.78	711.4	722.8	745.42	771.23	796.85
AA		P	T	L	Q	Q	I	S	H	I	A	Q	Q	L	G	L	E	K
a common y standard	+1	1904.06	1807.01	1705.96	1592.88	1464.82	1336.76	1223.67	1136.64	999.58	886.5	815.46	687.4	559.35	446.26	389.24	276.16	147.11
	+2	952.53	904.01	853.48	796.94	732.91	668.88	612.34	568.83	500.3	443.75	408.24	344.21	280.18	223.63	195.12	138.58	74.06
	+3	635.36	603.01	569.33	531.63	488.94	446.26	408.56	379.55	333.87	296.17	272.49	229.81	187.12	149.43	130.42	92.72	49.71
a xlink y standard	+2	-	-	-	-	-	-	-	-	-	-	-	-	-	-	-	-	-
	+3	-	-	-	-	-	-	-	-	-	-	-	-	-	-	-	-	-
	+4	-	-	-	-	-	-	-	-	-	-	-	-	-	-	-	-	-
	+5	-	-	-	-	-	-	-	-	-	-	-	-	-	-	-	-	-

β chain

		G	G	K	G	L	G	K
b common b standard	+1	58.03	115.05	-	-	-	-	-
	+2	29.52	58.03	-	-	-	-	-
	+3	20.01	39.02	-	-	-	-	-
b xlink b standard	+2	-	-	1812.99	1841.5	1898.04	1926.56	1990.6
	+3	-	-	1209	1228	1265.7	1284.71	1327.4
	+4	-	-	907	921.26	949.53	963.78	995.81
	+5	-	-	725.8	737.21	759.82	771.23	796.85
AA		G	G	K	G	L	G	K
b common y standard	+1	-	-	-	374.24	317.22	204.13	147.11
	+2	-	-	-	187.62	159.11	102.57	74.06
	+3	-	-	-	125.42	106.41	68.72	49.71
b xlink y standard	+2	1999.61	1971.1	1942.59	-	-	-	-
	+3	1333.41	1314.4	1295.39	-	-	-	-
	+4	1000.31	986.05	971.8	-	-	-	-
	+5	800.45	789.04	777.64	-	-	-	-

Supplementary Figure S6. Spectra of crosslinked peptides and table with matched fragment ion masses. (a) MS/MS spectra and fragment ion tables of the crosslinked peptides with the highest Ld-scores in OCT4-H3.1. **(b)** OCT4-H2A and **(c)** OCT4-H4 are depicted. Color coding in tables: green indicates a common fragment ion, and red indicates a crosslinker containing a fragment ion.

a

```
5’ ATCGGACCCTATCGCGAGCCAATTAACATAATCCGGTGCCGAGGCCGCTCAATTGGTCGTAGAC
3’ TAGCCTGGGATAGCGCTCGGTTAATTGTATTAGGCCACGGCTCCGGCGAGTTAACCAGCATCTG

   AGCTCTAGCACCGCTTAAACGCACGTACGCGCTGTCCCCCGCGTTTTAACCGCCAAGGGGATTA
   TCGAGATCGTGGCGAATTTGCGTGCATGCGCGACAGGGGGCGCAAAATTGGCGGTTCCCCTAAT

   CTCCCTAGTCTCCAGGCACGTGTCAGATCGGATTCTCCAGGCCTTGTGTCGCGAAGCGCAGCGAT 3’
   GAGGGATCAGAGGTCCGTGCACAGTCTAGCCTAAGAGGTCCGGAACACAGCGCTTCGCGTCGCTA 5’
```

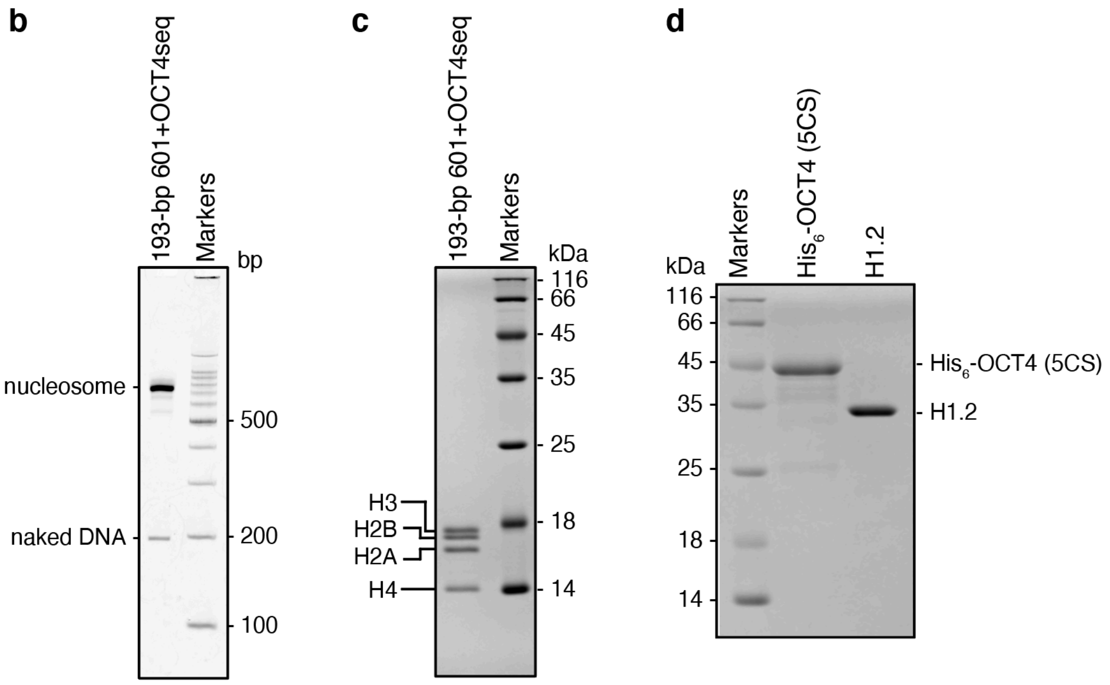

Supplementary Figure S7. Purification of the 601 nucleosome containing the OCT4 target DNA sequence. (a) Design of the 193 base-pair 601 DNA with the OCT4 target sequence. Grey highlighting indicates the 601 DNA region directly contacting the histone octamer in the nucleosome. The bases containing the OCT4 target DNA sequence are colored magenta. **(b,c)** Purification of the 193 base-pair 601 nucleosome with the OCT4 target sequence. The purified nucleosome was analyzed by non-denaturing polyacrylamide gel electrophoresis with EtBr staining **(b)** and by SDS-polyacrylamide gel electrophoresis with Coomassie Brilliant Blue staining **(c)**. **(d)** The purified His_6-tagged human OCT4 (5CS) protein (2.0 µg) and H1.2 (2.0 µg) were analyzed by SDS-polyacrylamide gel electrophoresis with Coomassie Brilliant Blue staining.

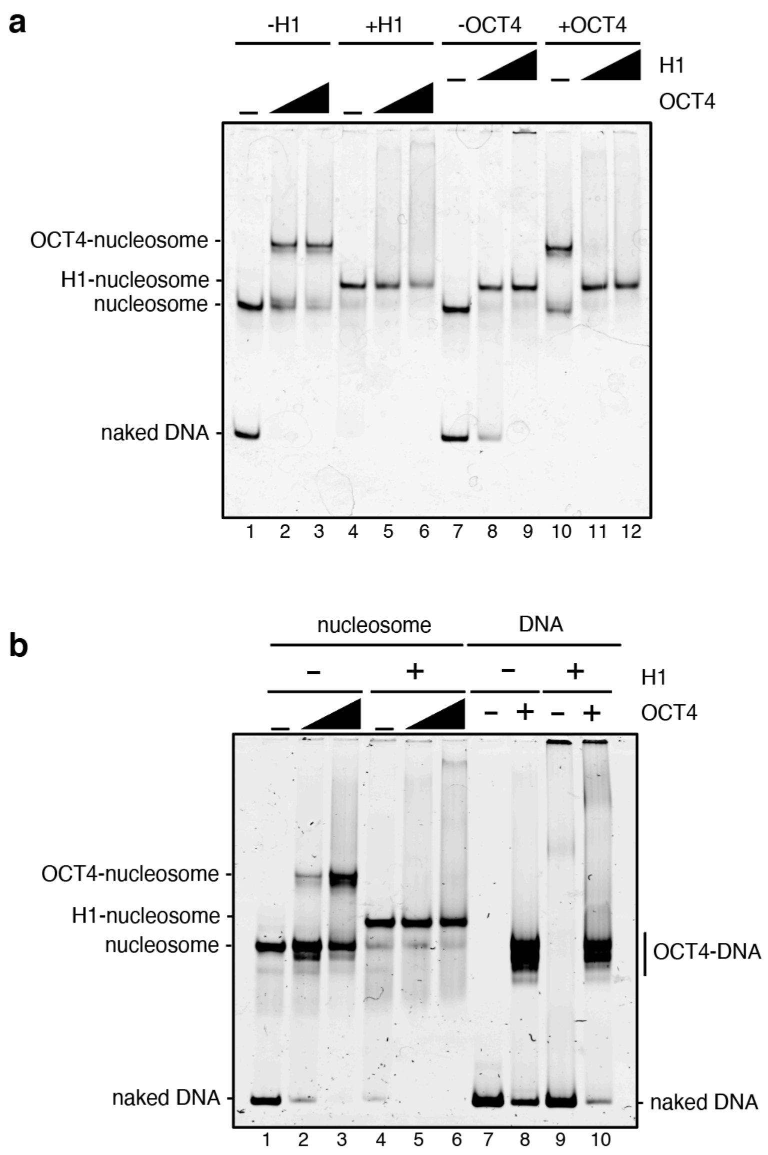

Supplementary Figure S8. Replicated experiments for Fig. 4b. (a) For the OCT4 titration, the nucleosome (0.1 μM) was preincubated with or without the linker histone H1.2 (0.9 μM), followed by the addition of 0 μM (lanes 1 and 4), 0.15 μM (lanes 2 and 5), and 0.3 μM (lanes 3 and 6) of OCT4. For the H1 titration, the nucleosome (0.1 μM) was preincubated with or without OCT4 (0.3 μM), followed by the addition of 0 μM (lanes 7 and 10), 0.45 μM (lanes 8 and 11), and 0.9 μM (lanes 9 and 12) of H1.2. Samples were analyzed by non-denaturing polyacrylamide gel electrophoresis with ethidium bromide staining. **(b)** The nucleosome or the H1.2-nucleosome complex was titrated with OCT4 (lanes 1-3 and 4-6, respectively). Naked DNA with the same sequence as the nucleosomal DNA was titrated with or without OCT4 and H1.2 (lanes 7-10). Nucleosome and DNA concentrations were 0.1 μM. For the nucleosome experiments, the OCT4 concentrations were 0 μM, 0.15 μM, and 0.3 μM. For the naked DNA experiments, the OCT4 concentrations were 0 μM and 0.3 μM. The H1.2 concentrations were 0 μM and 0.9 μM. Samples were analyzed by non-denaturing polyacrylamide gel electrophoresis with ethidium bromide staining.

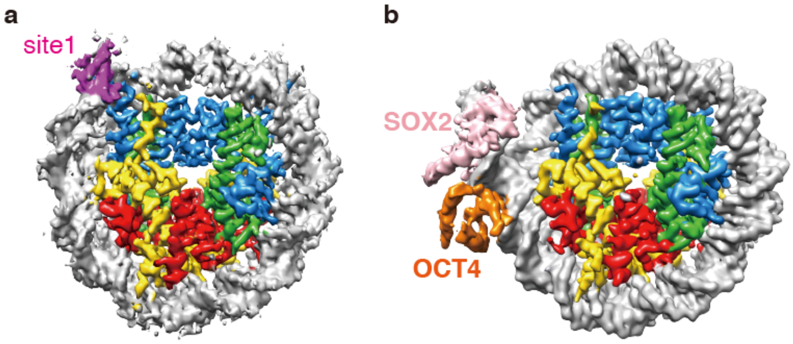

Supplementary Figure S9. Structural comparison between the LIN28B nucleosome and the OCT4-SOX2-nucleosome complex with a designed 601 DNA sequence. (a) Cryo-EM structure of the LIN28B nucleosome. Site 1 on the *LIN28B* distal enhancer DNA is colored magenta. **(b)** Cryo-EM structure of the OCT4-SOX2-nucleosome complex with a designed 601 DNA sequence (EMD-10406). OCT4 and SOX2 bound to their target sequences are colored orange and pink, respectively.

Table S1. Cryo-EM data collection and reconstruction

Sample	LIN28B nucleosome (EMD-30070)
**Data collection**	
Magnification	x100,000
Voltage (kV)	200
Pixel size (Å/pix)	1.32
Defocus range (μm)	-1.0 to -2.5
Exposure time (second)	10
Total dose (e/Å^2)	~50
Movie frames (no.)	40
Total micrographs (no.)	1,877
**Reconstruction**	
Software	Relion 3.0
Particles for 2D classification (no.)	325,272
Particles for 3D classification (no.)	264,297
Particles in the final map (no.)	150,721
Symmetry	C2
Final resolution (Å)	3.6
FSC threshold	0.143
Map sharpening B factor (Å^2)	-50.72
